# WBNPMD: weighted bipartite network projection for microRNA-disease association prediction

**DOI:** 10.1186/s12967-019-2063-4

**Published:** 2019-09-23

**Authors:** Guobo Xie, Zhiliang Fan, Yuping Sun, Cuiming Wu, Lei Ma

**Affiliations:** 1 School of Computer Science, Guangdong University of Technology, Guangzhou, China; 2 Institute of Automation, Chinese Academy of Sciences, Beijing, China

**Keywords:** miRNA-disease association, Bipartite network projection, Transfer weight assignment, Initial information configuration

## Abstract

**Background:**

Recently, numerous biological experiments have indicated that microRNAs (miRNAs) play critical roles in exploring the pathogenesis of various human diseases. Since traditional experimental methods for miRNA-disease associations detection are costly and time-consuming, it becomes urgent to design efficient and robust computational techniques for identifying undiscovered interactions.

**Methods:**

In this paper, we proposed a computation framework named weighted bipartite network projection for miRNA-disease association prediction (WBNPMD). In this method, transfer weights were constructed by combining the known miRNA and disease similarities, and the initial information was properly configured. Then the two-step bipartite network algorithm was implemented to infer potential miRNA-disease associations.

**Results:**

The proposed WBNPMD was applied to the known miRNA-disease association data, and leave-one-out cross-validation (LOOCV) and fivefold cross-validation were implemented to evaluate the performance of WBNPMD. As a result, our method achieved the AUCs of 0.9321 and $0.9173 \pm 0.0005$ in LOOCV and fivefold cross-validation, and outperformed other four state-of-the-art methods. We also carried out two kinds of case studies on prostate neoplasm, colorectal neoplasm, and lung neoplasm, and most of the top 50 predicted miRNAs were confirmed to have an association with the corresponding diseases based on dbDeMC, miR2Disease, and HMDD V3.0 databases.

**Conclusions:**

The experimental results demonstrate that WBNPMD can accurately infer potential miRNA-disease associations. We anticipated that the proposed WBNPMD could serve as a powerful tool for potential miRNA-disease associations excavation.

**Electronic supplementary material:**

The online version of this article (10.1186/s12967-019-2063-4) contains supplementary material, which is available to authorized users.

## Background

MiRNAs are a class of the short endogenous non-coding RNAs (ncRNAs), and their length are about 20–25 nucleotides [1]. These miRNAs can bind to specific target messenger RNAs (mRNAs), triggering regulated degradation or suppressing their translation [1–4]. In this way, various important biological processes are influenced by miRNAs, including cell development [5], proliferation [6], apoptosis [7], differentiation [8], metabolism [9, 10], aging [9, 10], and signal transduction [11]. In 2005, Croce and Calin discovered that the differential expression of miRNAs has a great influence on the development of various cancer [12], such as breast cancer [13], lung cancer [14], and prostate cancer [15]. Therefore, scientists devoted themselves to mining the disease-associated miRNAs in recent years, to have a better comprehension of the mechanism of diseases on the molecular level, and thus improve the disease diagnosis and treatment [16–18]. In the early stage of miRNA research, the identification of disease–miRNA associations was conducted by biological experiments, which are rather expensive and time-consuming. Therefore, increasing numbers of computational methods were developed into usage in the field of bioinformatics. Guided by the prediction result, miRNA-disease pairs with high potential uncovered by biological experiments were much more effective than before.

According to previous researches, miRNAs that have functional similarity regulates similar diseases and vice versa [19, 20]. Thus, various computational methods were developed for potential miRNA-disease associations excavation based on this assumption. So far, methods for miRNA-disease associations prediction can be roughly summarized into two categories, machine learning methods and complex network-based methods.

Generally, machine learning methods utilize the biological features of miRNA and disease to train classifiers for miRNA-disease associations prediction. So far, supervised and semi-supervised methods were widely employed for associations identification, and their difference lies in the requirement of negative samples in the training stage. In the supervised method presented by Xu et al. a support vector machine (SVM) classifier was trained by utilizing the topological information of miRNA target-dysregulated network (MTDN) for positive associations identification [21]. However, high confidence negative samples are very hard to obtain, which significantly influences the accuracy of a supervised classifier. Considering this factor, many semi-supervised methods were proposed by latter studies. For example, Chen and Yan [19] proposed a global method named RLSMDA based on regularized least squares. The RLSMDA could predict novel miRNA-disease associations without utilizing negative sample sets. Later, the GRMDA method proposed by Chen et al. [22] performed graph regression technique in three different latent spaces to infer potential miRNA-associated diseases. Recently, the IMCMDA proposed by Chen et al. [23] completed the missing miRNA-disease associations based on the known miRNA and disease similarity information. Another method proposed by Zhao et al. [24] namely NRLMFMDA focuses on the prediction task by mapping a miRNA and a disease to a shared low dimensional latent space. By using the L2 regularization to produce a finally optimized non-sparse combination of multiple base kernel, the MKRMDA proposed by Chen et al. [25] obtained a high prediction accuracy. Although these semi-supervised methods no longer require negative samples, their performance is unstable. In conclusion, the machine learning methods obtained an excellent result in miRNA-disease associations prediction.

By extracting information from the known miRNA-disease association network, complex network-based method offered an alternative approach in this field. There are two key factors for proposing network-based methods, the introduction of novel similarity information and different network construction techniques. With the fast development of biological research, more and more miRNA and disease similarity information became available, thus increasing numbers of studies started to introduce these novel information in their methods. The prediction accuracy can possibly be improved if these similarity information is made good use of, and the key lies in the construction technique of the miRNA-disease association network. Considering that the prediction accuracy of similarity measurement in the local network was unsatisfying [16], latter studies introduced many global network methods [26–29]. By implementing a random walk with restart into miRNA functional similarity network, Chen et al. developed the RWRMDA method for associations prediction [30]. With a given starting seed node, it simulates the process of the walker transfer from the current node to its neighborhood. However, the drawback of RWRMDA is that it could not predict new miRNA-disease pairs. The HDMP method proposed by Xuan et al. [31] employed the K-Nearest Neighbors technique to complete the prediction, which inspired many latter methods. Later, Liu et al. [32] calculated miRNA similarity based on miRNA-target and miRNA-lncRNA associations. Then a heterogeneous network was constructed by integrating known miRNA and disease information. Similarly, Luo and Xiao [33] implemented the unbalanced bi-random walk on a heterogeneous network. The HlPMDA proposed by Chen et al. also constructed a heterogeneous network, and implemented a heterogeneous label propagation to infer possible association [34]. By incorporating miRNA and disease similarity information, Jiang et al. [35] proposed an improved collaborative filtering algorithm. Recently, Chen et al. proposed a bipartite network projection model named BNPMDA [36]. By integrating known miRNA and disease similarity information, the BNPMDA constructed a weighted bipartite network, then the two-round resource allocation was implemented to uncover miRNA-disease associations.

According to previous works, network-based methods generally yield a higher prediction accuracy compared to machine learning methods, while the appropriate utilization of miRNA and disease similarities could further improve performance. In addition, the technique of assigning transfer weight to bipartite network model is widely employed to many research fields, and according to the study of Zhou et al. [37] the optimization of initial information in the bipartite network could bring extra benefit for improving prediction accuracy. Inspired by the aforementioned discussion, we proposed a novel method called weighted bipartite network projection for miRNA-disease association prediction (WBNPMD). In WBNPMD, the transfer weights in the bipartite network are assigned by combining known miRNA and disease similarities, and the initial information is properly configured by reducing the recommendation power of popular nodes. Compared to the previous machine learning methods, our method does not need negative samples. With the assignment of transfer weight and the configuration of initial information, our method acquired an even better result compared to other network-based methods. To evaluate the prediction accuracy of WBNPMD, we implemented leave-one-out cross-validation (LOOCV) and fivefold cross-validation on our collected dataset downloaded from HMDD V2.0 [38], obtaining the AUCs of 0.9321 and $0.9173 \pm 0.0005$. As an approach to further validation, we employed two types of case studies on three vital human diseases. These results indicated that our proposed method is a powerful tool for uncovering potential miRNA-disease associations.

## Methods

### Human miRNA-disease associations

In this article, we downloaded the known human miRNA-disease associations from HMDD v2.0 database, including 5430 associations, 383 diseases and 495 miRNAs. Also, the number of miRNA and disease are represented as *nm* and *nd* respectively. In order to formalize these associations, a adjacency matrix *A* is constructed. If disease $d_j$ has confirmed relation with miRNA $m_i$, then $A_{ij}$ is set to 1, otherwise 0.

### MiRNA functional similarity

According to the assumption that functionally similar miRNAs tend to related with phenotypically similar diseases, Wang et al. [39] proposed a calculation method for miRNAs functional similarity, and its scores is obtained from http://www.cuilab.cn/files/images/cuilab/misim.zip. A *nm* by *nm* matrix *FS* is constructed to represent miRNA functional similarity. Then the similarity score between two miRNAs $m_i$ and $m_j$ is denoted as *FS*(*i*, *j*).

### Disease semantic similarity model 1

Here, we will introduce two models for disease semantic similarity calculation. Based on the Medical Subject Headings (MeSH) descriptors, Wang et al. developed the first model [39]. Given a specific disease S, Directed Acyclic Graph (DAG) can be utilized for its representation, i.e. $DAG(S)=(S,T(S),E(S))$, where *T*(*S*) and *E*(*S*) denote the node set and edge set respectively. The contribution value of disease *t* in *DAG*(*S*) is defined as follows:

1 $$\begin{aligned} D1_S(t)=\left\{\begin{array}{ll} 1 & \quad if \ t=S\\ \max \{\Delta *D1_S (t^{\prime})|t^{\prime}\in \ children \ of \ t \} & \quad if \ t\ne S, \end{array} \right. \end{aligned}$$

where $\Delta$ is the semantic contribution decay parameter. The semantic value of disease *S* is defined as follows:

2 $$\begin{aligned} DV1(S)=\sum _{t\in T(S)} D1_S (t), \end{aligned}$$

where *T*(*S*) means all ancestor nodes of *S* and *S* itself. It is easy to conclude that the more DAG parts two diseases shared, the higher the semantic similarity score. Thus a *nd* by *nd* semantic similarity matrix *SS*1 is constructed, and entity *SS*1(*A*, *B*) representing the semantic similarity score between disease *A* and *B* can be defined as follows:

3 $$\begin{aligned} SS1(A,B)=\frac{\sum _{t\in T(A) \cap T(B) }(D1_{A}(t)+D1_{B}(t))}{DV1(A)+DV1(B)}, \end{aligned}$$

### Disease semantic similarity model 2

In disease similarity model 1, different ancestor diseases on the same layer of DAG(*S*) have same semantic contribution value. Considering that a more specific disease which appears in DAGs less frequently should have a higher contribution value to the semantic similarity of disease *S*, another disease semantic similarity model was proposed by Xuan et al. [31]. The contribution value of disease *S* in DAG(*S*) is defined as follows:

4 $$\begin{aligned} D2_S(t)=-\log \left( \frac{the \ number \ of \ DAGs \ including \ t}{the \ number \ of \ diseases} \right) . \end{aligned}$$

Based on model 2, the semantic similarity matrix *SS*2 is computed with the utilization of *DV*2(*A*) and *DV*2(*B*), and they are calculated by the same way as formula 2. Then the semantic similarity score *SS*2(*A*, *B*) between disease *A* and *B* can be calculated as follows:

5 $$\begin{aligned} SS2(A,B)=\frac{\sum _{t\in T(A) \cap T(B) }(D2_{A}(t)+D2_{B}(t))}{DV2(A)+DV2(B)}. \end{aligned}$$

At last, these two semantic similarity matrices *SS*1 and *SS*2 are combined into final semantic similarity matrix *SS* as follows:

6 $$\begin{aligned} SS(A,B)=\frac{SS1(A,B)+SS2(A,B)}{2}. \end{aligned}$$

### Gaussian interaction profile kernel similarity

As an another approach to measure miRNA similarity and disease similarity, Gaussian interaction profile kernel similarities were also be constructed using the Radial Basic Functions. In adjacency matrix A, the *i*th row means whether miRNA $m_i$ have associations with every disease, and the *j*th column means whether disease $d_j$ have associations with every miRNA. Vector $IP(m_i)$ and $IP(d_j)$ represent the *i*th row vector and the *j*th column vector as feature vector for Gaussian kernel. Thus, we defined the Gaussian interaction profile kernel similarity between diseases $d_i$ and $d_j$ as *KD*, the Gaussian interaction profile kernel similarity between miRNAs $m_i$ and $m_j$ as *KM*, and they can be calculated as follows:

7 $$\begin{aligned} KD(d_i,d_j)= & {} \exp {(-\beta _d||IP(d_i)-IP(d_j)||^2)}, \end{aligned}$$

8 $$\begin{aligned} KM(m_i,m_j)= & {} \exp {(-\beta _m||IP(m_i)-IP(m_j)||^2)}, \end{aligned}$$

Here, the kernel bandwidth $\beta _d$ and $\beta _m$ are defined as follows:

9 $$\begin{aligned} \beta _d = \beta ^{\prime}_d\left( \frac{1}{nd}\sum ^{n}_{i=1}||IP(d_i)||^2)\right) , \end{aligned}$$

10 $$\begin{aligned} \beta _m = \beta ^{\prime}_m\left( \frac{1}{nm}\sum ^{m}_{i=1}||IP(m_i)||^2)\right) . \end{aligned}$$

where we set the value of original kernel bandwidth parameters $\beta ^{\prime}_d$ and $\beta ^{\prime}_m$ to 1.

### Integrated similarity for miRNAs and diseases

From previous sections, we constructed several similarity matrices including miRNA functional similarity, disease semantic similarity and Gaussian profile kernel similarity. In here, we combined them into the integrated matrix for miRNAs and diseases. Concretely, if miRNA $m_i$ and $m_j$ are functionally similar, then the integrated similarity score for them is equal to $FS(m_i,m_j)$, otherwise is equal to $KM(m_i,m_j)$. The disease integrated matrix can be processed in a similar way. Then we computed the integrated matrices for miRNAs and diseases as follows:

11 $$\begin{aligned} MS(m_i,m_j) = \left\{\begin{array}{ll} FS(m_i,m_j), \quad m_i\ and\ m_j\ has\ functional\ similarity \\ KM(m_i,m_j), \quad otherwise, \end{array} \right. \end{aligned}$$

12 $$\begin{aligned} DS(d_i,d_j) = \left\{\begin{array}{ll} \frac{SS1(d_i,d_j)+SS2(d_i,d_j)}{2},\quad d_i\ and\ d_j\ has\ semantic\ similarity\\ KD(d_i,d_j), \quad otherwise. \end{array} \right. \end{aligned}$$

### WBNPMD

**Fig. 1:**
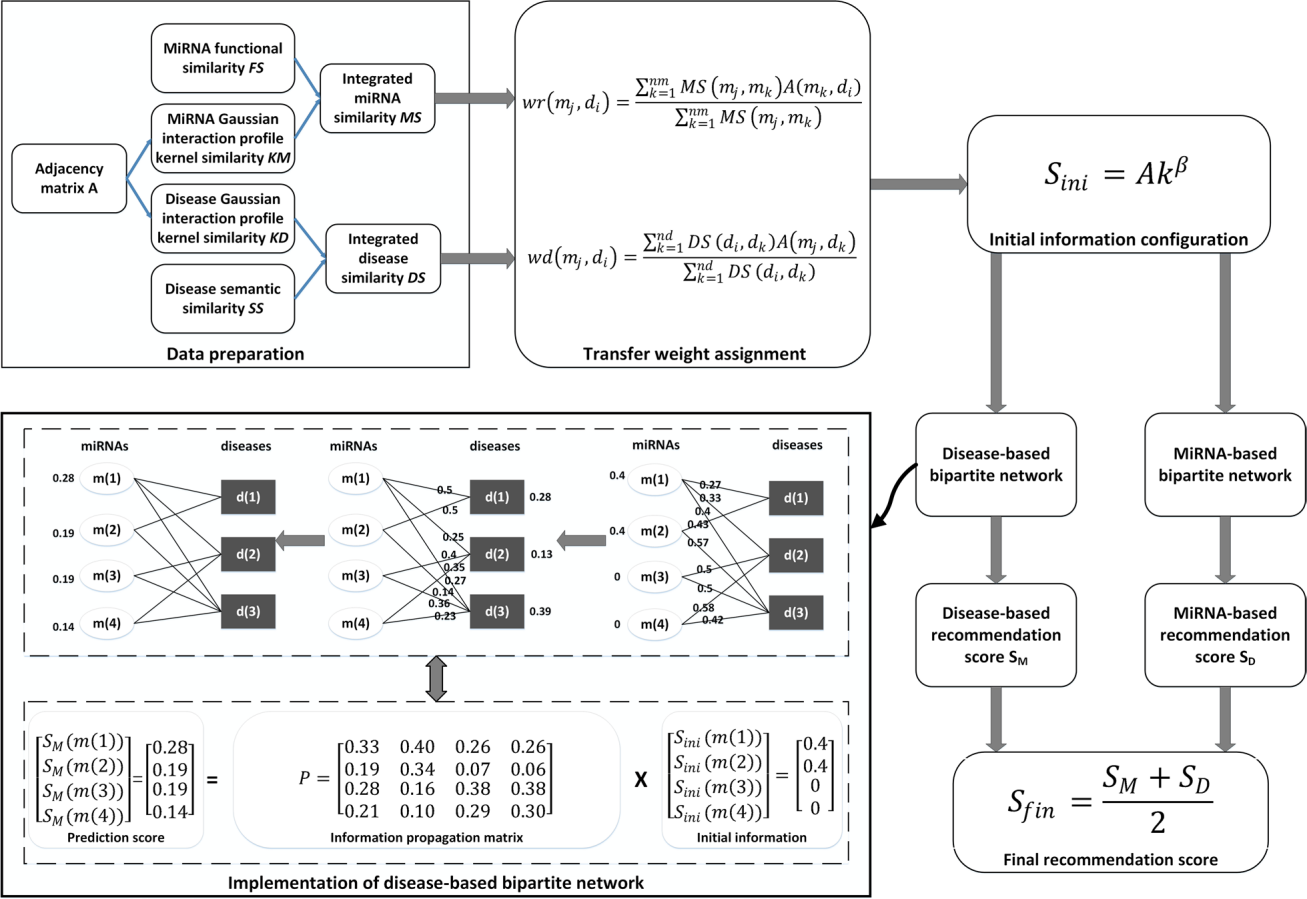
The basic idea of WBNPMD. In the first step, integrated similarity matrix are constructed by combining known miRNA-disease associations, miRNA and disease similarity information. Next, after the steps of transfer weight assignment and initial information configuration, two bipartite networks are constructed. Finally, the disease-based and miRNA-based bipartite network are separately implemented, and the final prediction result is obtained by averaging the recommendation score of above

In this paper, we presented a bipartite network based method for miRNA-disease associations prediction named WBNPMD. The data preparation process for WBNPMD has been presented from previous six sections. The flowchart of WBNPMD is shown in Fig. 1.

According to the assumption that similar miRNAs have higher chance to associate with similar diseases and vice versa, we utilized the integrated similarity of miRNA and disease to assign transfer weight to every edges in the miRNA-disease bipartite network. Therefore, the transfer weights are denoted as the following equation:

13 $$\begin{aligned} wr(m_j,d_i) = \frac{ \sum _{k=1} ^{nm} MS(m_j,m_k) A(m_k,d_i) }{ \sum _{k=1} ^{nm} MS(m_j,m_k) }, \end{aligned}$$

14 $$\begin{aligned} wd(m_j,d_i) = \frac{ \sum _{k=1} ^{nd} DS(d_i,d_k) A(m_j,d_k) }{ \sum _{k=1} ^{nd} DS(d_i,d_k) }, \end{aligned}$$

where $wr(m_j,d_i)$ is the transfer weight of the edge from miRNA $m_j$ to disease $d_i$, and $wd(m_j,d_i)$ is the transfer weight of the edge from disease $d_i$ to miRNA $m_j$. The transfer weight *wr* represents the recommendation power of every miRNA to different diseases, while *wd* represents the recommendation power of every disease to different miRNAs, indicating miRNA-disease pairs with higher potential.

We utilized known miRNA and disease similarity information to construct a more accurate bipartite network. Concretely, we separately implemented the disease-based bipartite network and the miRNA-based bipartite network. In the first implementation, all miRNAs are recommended to diseases, while in the second implementation all diseases are recommended to miRNAs. The recommendation score is obtained by averaging the final information matrices.

In the next, we will detailedly introduce the implementation of disease-based bipartite network. According to the study of Zhou et al. [37] reducing the initial information of popular nodes may lead to higher prediction accuracy. Therefore we denote the initial information between miRNA $m_j$ and disease $d_i$ as follows:

15 $$\begin{aligned} S_{ini}(m_j,d_i)=A_{ji}k_{i}^{\beta }, \end{aligned}$$

where $S_{ini}$ is the initial information matrix, $k_i$ is the number of miRNAs that associated with disease $d_i$, and parameter $\beta \in (-1,0)$.

After the initial information of all miRNAs and the transfer weight of every edges in the bipartite network are all set, we begin the information propagation process to obtain the final recommendation score. The information propagation process can be separated into two steps. In the first step, the initial information propagated from every miRNA to disease $d_i$ is calculated as:

16 $$\begin{aligned} S_{mid}(d_i)=\sum _{k=1} ^{nm} \frac{wr(m_k,d_i) S_{ini}(m_k,d_i)}{d(m_k)}, \end{aligned}$$

where

17 $$\begin{aligned} d(m_k)=\sum _{i=1} ^{nd} wr(m_k,d_i). \end{aligned}$$

In the second step, we propagate the information of diseases gathered from step one back to miRNAs to obtain the recommendation score, and can be calculated as the following equation:

18 $$\begin{aligned} S_M(m_j)=\sum _{i=1} ^{nd} \frac{wr(m_j,d_i) S_{mid}(d_i)}{d(d_i)} =\sum _{i=1} ^{nd} \frac{wr(m_j,d_i)}{d(d_i)} \sum _{k=1} ^{nm} \frac{wr(m_k,d_i) S_{ini}(m_k,d_i)}{d(m_k)}, \end{aligned}$$

where

19 $$\begin{aligned} d(d_i)=\sum _{j=1} ^{nm} wr(m_j,d_i). \end{aligned}$$

The disease-based recommendation score matrix $S_M$ can also be defined as follows:

20 $$\begin{aligned} {S_M}=P{S_{ini}}. \end{aligned}$$

Here, *P* is defined as the *nm* by *nm* propagation matrix, and ${S_M}$ is the recommendation score gathered by two-step information propagation of weighted miRNA-disease bipartite network. The entity $P(m_j,m_k)$ in propagation matrix *P*, which represents the information gathered by miRNA $m_j$ from $m_k$ is defined as follows:

21 $$\begin{aligned} P(m_j,m_k)=\frac{1}{d(m_k)} \sum _{i=1} ^{nd} \frac{wr(m_j,d_i) wr(m_k,d_i)}{d(d_i)}. \end{aligned}$$

Hence, equation 18 can also be rewritten as follows:

22 $$\begin{aligned} S_M(m_j)=\sum _{k=1} ^{m} P(m_j,m_k) S_{ini}(m_k,d_i), \end{aligned}$$

The equations from 15 to 22 are the details for the disease-based bipartite network. We similarly implemented the miRNA-based bipartite network to recommend diseases to miRNAs, and obtained the recommendation score matrix $S_D$ which represents the information propagated from diseases to miRNAs. Lastly, we calculated the final recommendation score matrix $S_{fin}$ between every miRNA-disease pairs by averaging $S_M$ and $S_D$ as follows:

23 $$\begin{aligned} S_{fin}=\frac{S_M+S_D}{2} \end{aligned}$$

## Results

### Evaluation metrics

To evaluate the performance of WBNPMD for miRNA-disease associations identification, the LOOCV and fivefold cross-validation techniques were performed on the collected dataset. In each trial of LOOCV, each known miRNA-disease associations were treated as a test sample in turn while the rest were taken as training samples. The receiver operating characteristic (ROC) curve was plotted to visualize the performance of WBNPMD, and the area under the ROC curve (AUC) was computed to illustrate the superiority of our method. In fivefold cross-validation, all known miRNA-disease associations were randomly divided into 5 groups with equal size. Each group was left out as a test sample in turn, while the other 4 groups were utilized for training. To avoid data bias, the fivefold cross-validation was repeated 100 times, then we computed the average AUC value.

### Effect of parameter

**Fig. 2:**
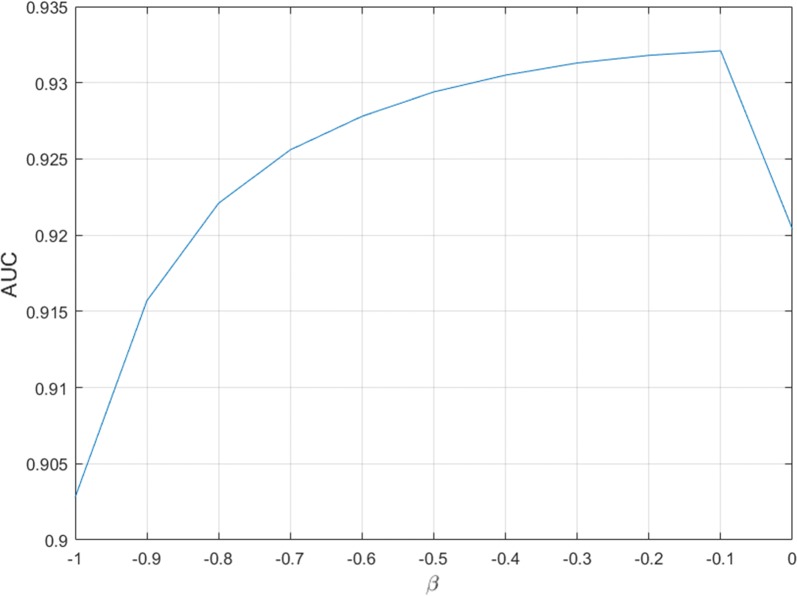
The AUCs of WBNPMD with different parameter choices of $\beta$

The WBNPMD method introduced one parameter $\beta$. According to Eq. (15), $\beta$ configures the initial information of every node in the bipartite network. To study the effect of $\beta$, the LOOCV technique was implemented in the miRNA-disease associations dataset to observe how different $\beta$ values would influence the AUCs. LOOCV was repeated multiple times by choosing the parameter value of $\beta$ from − 1 to 0 with the step of 0.1. As shown in Fig. 2, we can observe that the AUCs have little fluctuation in the parameter range from − 1 to 0. The optimal parameter $\beta$ is chosen based on the highest AUC value in the figure. In this paper, we set the parameter value of $\alpha$ to − 0.1.

### Performance comparison

**Fig. 3:**
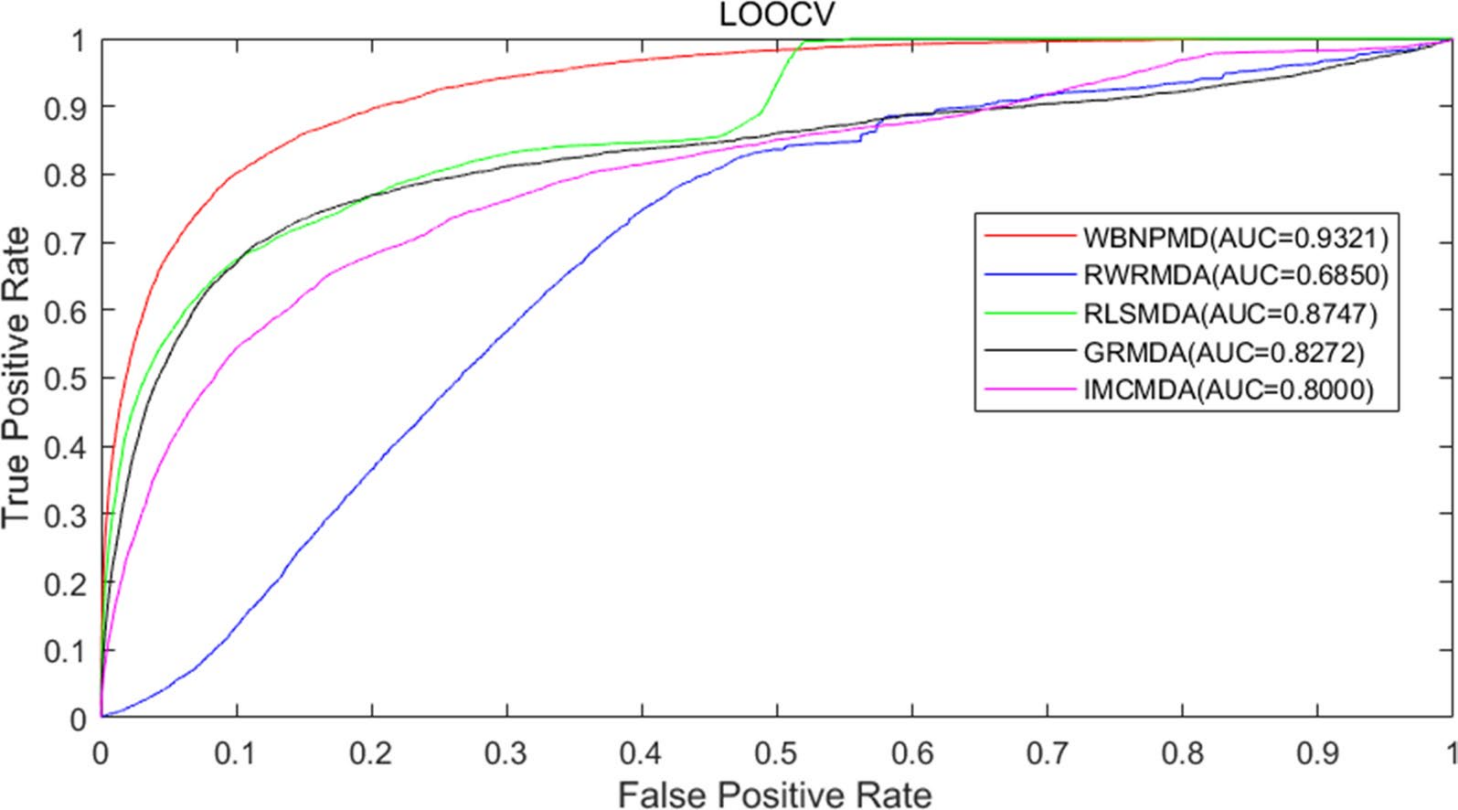
Performance comparison between WBNPMD and other four miRNA-disease association prediction models (RWRMDA, RLSMDA, GRMDA and IMCMDA) by means of ROC curves and AUCs based on LOOCV

**Fig. 4:**
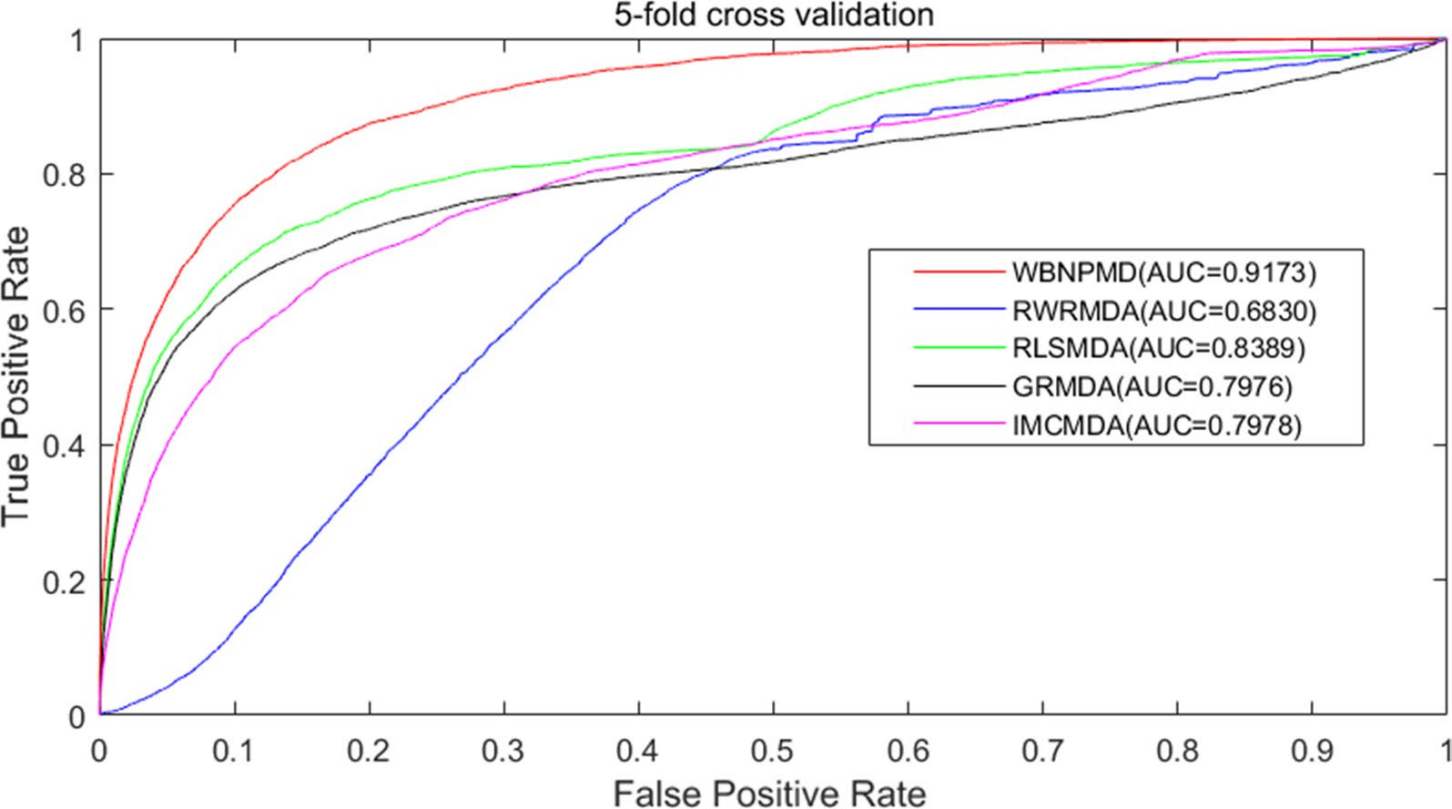
Performance comparison between WBNPMD and other four miRNA-disease association prediction models (RWRMDA, RLSMDA, GRMDA and IMCMDA) by means of ROC curves and AUCs based on fivefold cross-validation

In order to express the reliability of WBNPMD, we compared WBNPMD with other four state-of-the-art methods, including RWRMDA, RLSMDA, GRMDA, and IMCMDA. All these methods were reproduced by ourselves on the same collected dataset and were assessed by LOOCV and fivefold cross-validation. The result of LOOCV is shown in Fig. 3, WBNPMD achieved the highest AUC value of 0.9321, while the AUCs of RWRMDA, RLSMDA, GRMDA and IMCMDA were 0.6850, 0.8716, 0.8747 and 0.8272. The ROC curves of fivefold cross-validation are also represented in Fig. 4. To conclude, the AUCs of RWRMDA, RLSMDA, GRMDA and IMCMDA were $0.6830 \pm 0.0078$, $0.8389 \pm 0.0006$, $0.7976 \pm 00023$ and $0.7978 \pm 0.0014$ respectively, while WBNPMD produced the reliable AUC of $0.9173 \pm 0.0005$.

### Case studies

As an approach of further evaluation, three important human diseases were further verified through two types of case studies based on three different miRNA-disease databases named dbDEMC, miR2Disease and HMDD v3.0. We recorded the number of experimentally confirmed miRNAs in top 10, top 20, and top 50 that have associations with three diseases. In addition, the prediction result of all candidate miRNAs were publicly released for further expermental verification (see Additional file 1).

Prostate neoplasms are one of the most frequently diagnosed malignant tumor in men, resulting in increased morbidity and mortality with age [40, 41]. According to studies, some miRNAs could be the diagnostic biomarker for prostate neoplasms and even be helpful for the treatment process. For example, previous studies showed that miR-20 is vital to the regulation of prostate neoplasms [42], and upregulated expression of miR-483-5p would cause prostate cancer cell growth [43]. As shown in Table 1, 10 out of the top 10, 20 out of the top 20, and 47 out of the top 50 predicted miRNAs were experimentally confirmed to have an association with prostate neoplasms based on dbDEMC or miR2Disease.

**Table 1:** Prediction of the top 50 miRNAs associated with prostate neoplasms

miRNA	Evidence	miRNA	Evidence
hsa-mir-21	dbDEMC;miR2Disease	hsa-let-7b	dbDEMC;miR2Disease
hsa-mir-155	dbDEMC	hsa-mir-200c	dbDEMC
hsa-mir-146a	miR2Disease	hsa-mir-181a	dbDEMC
hsa-mir-17	dbDEMC	hsa-mir-200a	dbDEMC
hsa-mir-20a	dbDEMC;miR2Disease	hsa-let-7c	dbDEMC;miR2Disease
hsa-mir-34a	dbDEMC;miR2Disease	hsa-mir-210	dbDEMC;miR2Disease
hsa-mir-221	dbDEMC;miR2Disease	hsa-mir-34c	Unconfirmed
hsa-mir-92a	dbDEMC	hsa-mir-133a	dbDEMC
hsa-mir-126	dbDEMC;miR2Disease	hsa-mir-142	Unconfirmed
hsa-mir-16	dbDEMC;miR2Disease	hsa-mir-146b	dbDEMC
hsa-mir-18a	dbDEMC	hsa-mir-9	dbDEMC
hsa-mir-19b	dbDEMC;miR2Disease	hsa-mir-150	dbDEMC
hsa-mir-29a	dbDEMC;miR2Disease	hsa-mir-182	dbDEMC;miR2Disease
hsa-let-7a	dbDEMC;miR2Disease	hsa-mir-181b	dbDEMC;miR2Disease
hsa-mir-29b	dbDEMC;miR2Disease	hsa-mir-106b	dbDEMC
hsa-mir-19a	dbDEMC	hsa-let-7e	dbDEMC
hsa-mir-1	dbDEMC	hsa-mir-203	dbDEMC
hsa-mir-143	dbDEMC;miR2Disease	hsa-let-7d	dbDEMC;miR2Disease
hsa-mir-15a	dbDEMC;miR2Disease	hsa-mir-141	dbDEMC;miR2Disease
hsa-mir-200b	dbDEMC	hsa-mir-214	dbDEMC;miR2Disease
hsa-mir-222	dbDEMC;miR2Disease	hsa-mir-133b	dbDEMC
hsa-mir-223	dbDEMC;miR2Disease	hsa-let-7i	dbDEMC
hsa-mir-199a	dbDEMC;miR2Disease	hsa-let-7f	dbDEMC;miR2Disease
hsa-mir-29c	dbDEMC	hsa-mir-34b	Unconfirmed
hsa-mir-31	dbDEMC;miR2Disease	hsa-mir-196a	dbDEMC

Colorectal neoplasms are the third most common cancer type in both men and women with high a mortality rate, causing about 700,000 deaths every year. Only about 10% of colorectal neoplasms cases are hereditary, while most of the rest are posteriority. Studies confirmed that several factors may be the cause of colorectal neoplasms, including alcohol consumption, smoking, and physical inactivity [44]. Various miRNAs were confirmed to have a relation with colorectal neoplasms in recent researches. Take miR-10a for an example, by differently expressed in SW480 and SW620 cell lines, it could suppress the metastasis of colorectal cancer [45]. The proposed WBNPMD was employed on colorectal neoplasms and verified through dbDEMC and miR2Disease. As shown in Table 2, 10 out of the top 10, 19 out of the top 20, and 46 out of the top 50 miRNAs were experimentally confirmed.

**Table 2:** Prediction of the top 50 miRNAs associated with colorectal neoplasms

miRNA	Evidence	miRNA	Evidence
hsa-mir-15a	dbDEMC	hsa-mir-30d	dbDEMC
hsa-mir-29b	dbDEMC;miR2Disease	hsa-mir-302a	dbDEMC
hsa-mir-223	dbDEMC;miR2Disease	hsa-mir-196b	dbDEMC
hsa-let-29c	dbDEMC	hsa-mir-302c	dbDEMC
hsa-mir-7d	dbDEMC	hsa-mir-204	dbDEMC
hsa-mir-106b	dbDEMC;miR2Disease	hsa-mir-296	miR2Disease
hsa-let-7i	dbDEMC	hsa-mir-30e	dbDEMC
hsa-let-7f	dbDEMC	hsa-mir-10a	dbDEMC;miR2Disease
hsa-mir-214	dbDEMC	hsa-mir-98	dbDEMC
hsa-let-7g	dbDEMC;miR2Disease	hsa-mir-99b	dbDEMC
hsa-mir-24	dbDEMC	hsa-mir-212	dbDEMC
hsa-mir-101	dbDEMC	hsa-mir-302d	dbDEMC
hsa-mir-15b	dbDEMC;miR2Disease	hsa-mir-32	dbDEMC;miR2Disease
hsa-mir-205	Unconfirmed	hsa-mir-181c	dbDEMC
hsa-mir-125a	dbDEMC;miR2Disease	hsa-mir-153	dbDEMC
hsa-mir-100	dbDEMC	hsa-mir-130b	dbDEMC;miR2Disease
hsa-mir-30c	dbDEMC;miR2Disease	hsa-mir-424	dbDEMC
hsa-mir-132	dbDEMC;miR2Disease	hsa-mir-181d	dbDEMC
hsa-mir-30b	dbDEMC	hsa-mir-197	dbDEMC
hsa-mir-192	dbDEMC;miR2Disease	hsa-mir-449a	Unconfirmed
hsa-mir-20b	dbDEMC	hsa-mir-452	dbDEMC
hsa-mir-23b	dbDEMC	hsa-mir-138	dbDEMC
hsa-mir-302b	dbDEMC	hsa-mir-494	Unconfirmed
hsa-mir-193b	dbDEMC	hsa-mir-449b	Unconfirmed
hsa-mir-191	dbDEMC;miR2Disease	hsa-mir-383	dbDEMC

In the second type of case studies, we evaluated the prediction accuracy of WBNPMD in lung neoplasms based on HMDD V2.0 database, and our results were validated in HMDD V3.0, dbDEMC and miR2Disease. As the most common cancer in the world, lung cancer causes about 1.4 million deaths per year [46]. Based on the result given by Table 3, 10, 20 and 47 out of the top 10, 20 and 50 miRNAs were confirmed to have an association with lung neoplasms by the aforementioned three databases. Taken together, these case studies above have indicated that WBNPMD has an outstanding performance for uncovering potential miRNA-disease associations.

**Table 3:** Prediction of the top 50 miRNAs associated with lung neoplasms

miRNA	Evidence	miRNA	Evidence
hsa-mir-16	dbDEMC;miR2Disease;HMDD	hsa-mir-99b	dbDEMC
hsa-mir-15a	dbDEMC;HMDD	hsa-mir-367	dbDEMC
hsa-mir-106b	dbDEMC	hsa-mir-339	dbDEMC;miR2Disease
hsa-mir-141	dbDEMC;miR2Disease;HMDD	hsa-mir-302d	dbDEMC
hsa-mir-15b	dbDEMC	hsa-mir-215	dbDEMC;HMDD
hsa-mir-195	dbDEMC;miR2Disease;HMDD	hsa-mir-149	dbDEMC;HMDD
hsa-mir-122	dbDEMC;HMDD	hsa-mir-28	dbDEMC
hsa-mir-429	dbDEMC;miR2Disease	hsa-mir-129	dbDEMC;HMDD
hsa-mir-20b	dbDEMC	hsa-mir-139	dbDEMC;miR2Disease;HMDD
hsa-mir-23b	dbDEMC	hsa-mir-153	dbDEMC;HMDD
hsa-mir-130a	dbDEMC;miR2Disease;HMDD	hsa-mir-130b	dbDEMC;HMDD
hsa-mir-373	dbDEMC;HMDD	hsa-mir-424	dbDEMC
hsa-mir-302b	dbDEMC	hsa-mir-181d	dbDEMC
hsa-mir-193b	dbDEMC	hsa-mir-491	dbDEMC
hsa-mir-302a	dbDEMC	hsa-mir-451a	dbDEMC;HMDD
hsa-mir-194	dbDEMC;HMDD	hsa-mir-144	dbDEMC;HMDD
hsa-mir-196b	dbDEMC;HMDD	hsa-mir-452	dbDEMC
hsa-mir-99a	dbDEMC;miR2Disease;HMDD	hsa-mir-449a	dbDEMC;HMDD
hsa-mir-302c	dbDEMC	hsa-mir-378a	Unconfirmed
hsa-mir-92b	dbDEMC	hsa-mir-148b	dbDEMC
hsa-mir-204	dbDEMC;miR2Disease	hsa-mir-449b	dbDEMC;HMDD
hsa-mir-342	dbDEMC;HMDD	hsa-mir-520b	dbDEMC;HMDD
hsa-mir-296	Unconfirmed	hsa-mir-151a	Unconfirmed
hsa-mir-10a	dbDEMC;HMDD	hsa-mir-383	dbDEMC
hsa-mir-372	dbDEMC;HMDD	hsa-mir-184	dbDEMC;HMDD

## Discussion

The results from above illustrate that both in LOOCV and fivefold cross-validation, the WBNPMD outperforms other comparison methods in terms of AUC. In addition, two types of case studies further confirmed the excellent performance of our proposed method. The excellent performance of WBNPMD can mainly be attributed to two reasons, the construction of transfer weight in the bipartite network and the adjustment of initial information. By combining known miRNA similarities and disease similarities, the weighted bipartite network is suitable for our work, guaranteeing a more precise result. Meanwhile, decreasing the initial information of popular nodes can further improve the prediction accuracy.

However, our method still has some limitations. First of all, the information completeness of the adjacency matrix *A* will have a heavy impact on the performance of WBNPMD. Moreover, the bipartite network projection model that we employ for predicting potential miRNA-disease associations cannot deal with the isolated nodes,^1^ thus WBNPMD is not suitable for the excavation of the associations for a miRNA without any known associated disease or vice versa.

## Conclusions

In this paper, we proposed the weighted bipartite network projection for miRNA-disease prediction (WBNPMD) method. LOOCV and fivefold cross-validation techniques were implemented to evaluate the performance of WBNPMD based on our collected dataset. The AUC values of the WBNPMD was 0.9321 in LOOCV and $0.9173 \pm 0.0005$ in fivefold cross-validation. Also, two types of case studies were conducted by implementing WBNPMD on three important human diseases. As a result, 47 (prostate neoplasms), 46 (colorectal neoplasms) and 47 (lung neoplasms) out of the top 50 predicted miRNAs were experimentally confirmed. All the results from above indicate that WBNPMD is a power tool for novel miRNA-disease association prediction.

## Supplementary information

**Additional file 1.** All potential miRNA-disease associations were ranked by WBNPMD utilizing data obtained from HMDDv2.0. Prediction results were publicly released for future study.

## Data Availability

The source codes and datasets used in this work could be freely downloaded at https://github.com/Dicrop/WBNPMD.

## Abbreviations

miRNA: microRNA
LOOCV: leave-one-out cross-validation
ROC: receiver operating characteristics curve
AUC: the area under ROC curve

## Footnotes

1 On the bipartite network, we treat a miRNA or a disease as a node. An isolated node implies that the miRNA do not have a confirmed link to a disease or vice versa.
